# White matter quantitative anomalies and clinical outcome in drug-resistant epilepsies

**DOI:** 10.1093/psyrad/kkaf015

**Published:** 2025-05-28

**Authors:** Edwin Humberto Hodelin Maynard, Nelson Ernesto Quintanal Cordero, Zenaida Milagros Hernández Díaz, Martha Caridad Ríos Castillo, Lilia María Morales Chacón

**Affiliations:** Transilvania University of Brasov, Faculty of Medicine, Transilvania Neurogenetic, Brașov, PC 500019, Romania; International Centre of Neurological Restoration, Neurosurgery Department, Havana, PC 11300, Cuba; CENIT Foundation for Research in Neurosciences, Autonomous City of Buenos Aires, PC C1125ABD, Argentina; International Centre of Neurological Restoration, Diagnostic Department, Havana, PC 11300, Cuba; International Centre of Neurological Restoration, Neurosurgery Department, Havana, PC 11300, Cuba; International University of Rioja, Neuropsychology and Education Department, Logroño, PC 26006, Spain; Clinical Hospital of Chengdu Brain Science Institute, MOE Key Lab for Neuroinformation, University of Electronic Science and Technology of China, Chengdu, PC 610218, China

**Keywords:** epilepsy surgery, automated fiber quantification, neurosurgery, functional neurosurgery, drug-resistant epilepsy

## Abstract

**Background:**

We aimed to evaluate the relationship between cerebral white matter quantitative anomalies and postoperative clinical outcomes in patients with drug-resistant epilepsies.

**Methods:**

Automatic fiber quantification methodology was used to determine the diffusional anomalies in cerebral white matter tracts, from eight patients with frontal lobe epilepsy (FLE) and 12 with temporal lobe epilepsy (TLE) candidates and/or undergoing epilepsy surgery. We studied the fractional anisotropy (FA), mean diffusivity (MD), volume (Vol), and number of fibers (Fib) of white matter tracts related to the physiopathologic mechanism of these epilepsies. The information was compared with that obtained from 19 healthy controls and between patients with seizure freedom and those with seizure recurrence 1 year after epilepsy surgery.

**Results:**

Significant pre- and postsurgical global and segmental abnormalities were characterized by increased MD and decreased FA, Vol, and Fib in tracts from both hemispheres. TLE patients with postsurgical seizure freedom had preoperative increased global MD of the contralateral inferior longitudinal fasciculus and uncinate fasciculus. Furthermore, drug-resistant epilepsy patients with seizure freedom had a presurgical segmental increased MD in the contralateral thalamic radiation. Additionally, FLE patients with seizure freedom exhibited postsurgical increases in the Fib of the ipsilateral thalamic radiation and contralateral inferior longitudinal fasciculus. Furthermore, temporal lobe epilepsy patients with seizure freedom had a postsurgical lower global MD in the ipsilateral inferior fronto-occipital fasciculus.

**Conclusions:**

Patients with drug-resistant epilepsies have global and segmental quantitative white matter tract anomalies, which suggests cerebral neural involvement in this disease. These abnormalities can vary regarding the postsurgical clinical outcome.

## Graphical Abstract

**Graphical Abstract:**
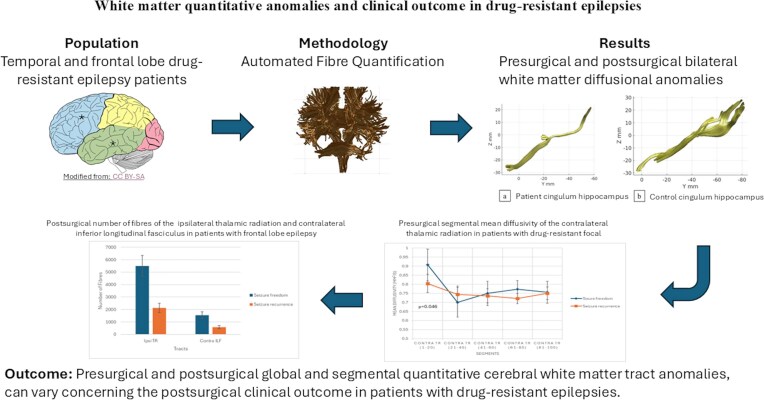

## Introduction

Epilepsy is among the most common neurological disorders, affecting over 50 million people across the world (WHO, n.d.).

Up to 30% of all epilepsy patients do not have remission of seizures despite appropriate therapy with antiseizure medications (ASMs). This condition is known as drug-resistant epilepsy (DRE). It is defined as the failure of adequate trials of two tolerated and appropriately chosen and used ASM schedules (whether as monotherapies or in combination) to achieve sustained seizure freedom (Kwan and Brodie, 2000; Kwan *et al*., 2009).

In addition, 5–10% of all patients diagnosed with DRE may be candidates for epilepsy surgery. Patients with temporal lobe epilepsy (TLE) account for 80% of surgical candidates. In contrast, patients with frontal lobe epilepsy (FLE) represent the majority of extratemporal surgery cases (Morales Chacón, 2017; Comair *et al*., 2022). As a result, surgical resection provides up to 70% seizure remission in TLE and 50–55% in extratemporal epilepsy (Morales Chacón *et al*., 2021; Brahimaj *et al*., 2023).

In this context, the study of epileptic patients using magnetic resonance imaging (MRI) has facilitated the understanding of this disease. The information provided by diffusion sequences on MRI can be used by diffusion tensor imaging (DTI) techniques. This allows the white matter tracts of the brain to be reconstructed utilising different deterministic and probabilistic tractography techniques (Keller *et al*., 2017).

Deterministic tractography traces fibers by following the principal direction of diffusion (usually the major eigenvector of the diffusion tensor) from voxel to voxel, generating a single, reproducible path per seed point. In contrast, probabilistic tractography accounts for uncertainty in fiber orientation by sampling multiple possible directions from a distribution at each voxel, resulting in a map of connection probabilities rather than a single path (Lilja *et al*., 2014).

Automated Fibre Quantification (AFQ) is a program which relies on deterministic methods for tractography. It may incorporate probabilistic elements in post-processing steps, such as quantifying tract properties or assessing variability across subjects. However, the core tractography process itself is deterministic (Yeatman *et al*., 2012).

These characteristics allow AFQ to identify multiple white matter tracts. In addition, the program segments each tract into 100 parts and measures different quantitative variables in each one (Yeatman, 2024).

Consequently, a comprehensive analysis of tissue characteristics along the entire length of white matter tract bundles can be achieved with AFQ. Furthermore, it provides a more precise measurement of neuroanatomical white matter alterations than whole-tract approaches. This is because it considers regional intra-tract tissue characteristics (Keller *et al*., 2017).

Previous studies on cerebral connectivity in TLE and FLE patients have detected diffusivity abnormalities in various white matter tract, including: the fornix, uncinate fasciculus (Un), inferior fronto-occipital fasciculus (IFOF), inferior longitudinal fasciculus (ILF), thalamic radiation (TR), forceps minor of the corpus callosum (CFMe), and the temporal part of the cingulum known as the cingulum hippocampus (CH) (Campos *et al*., 2015; Keller *et al*., 2017).

These findings indicate that focal epilepsy is a neural network disease affecting the white matter of the brain (Whelan *et al*., 2018). Furthermore, this information helps to understand why some patients with drug-resistant focal epilepsies do not have a favorable post-operative clinical outcome. It should be noted that this can occur even though the patients have been evaluated as ideal candidates for epilepsy surgery(Hodelin-Maynard *et al*., 2023).

In patients diagnosed with drug-resistant TLE and FLE, it is likely that some white matter abnormalities are present in specific tract regions, rather than along the entire tract. In this context, we also consider that AFQ is a suitable option for the study of these types of DRE.

Consequently, we hypothesize that specific white matter quantitative abnormalities, as identified by AFQ, are associated with postoperative clinical outcomes in patients with TLE and FLE undergoing epilepsy surgery.

Our investigation aims to evaluate the relationship between quantitative anomalies in cerebral white matter and postsurgical clinical outcomes in patients with drug-resistant focal epilepsies.

The potential detection of such anatomical specificities could improve the identification of anatomical prognostic markers of clinical outcome after surgical treatment in patients with DRE. This could also lead to greater accuracy in patient assessment and/or management.

## Materials and methods

A retrospective, observational, analytical, single-center, quantitative study was conducted on adolescent and adult patients who were evaluated and/or underwent epilepsy surgery for FLE and TLE at the International Centre for Neurological Restoration (CIREN) in Havana between January 2017 and December 2020.

Inclusion criteria comprised: adolescent and adult patients with drug-resistant FLE or TLE who were assessed and/or underwent epilepsy surgery; a minimum postoperative period of 12 months for evaluation of clinical outcomes; and presurgical and postsurgical assessment with 1.5T brain MRI with epilepsy protocol (Bernasconi *et al*., 2019).

Those patients in whom the technical analysis of diffusion-weighted imaging (DWI) obtained by a 1.5T brain MRI with epilepsy protocol (Bernasconi *et al*., 2019) was not completed, and individuals who did not complete the postsurgical clinical follow-up for the multidisciplinary team of CIREN were excluded from the study.

The total population comprised 20 patients, 12 subjects with TLE and eight with FLE. Of the patients in question, 10 individuals with TLE and seven with FLE underwent epilepsy surgery, performed by the same surgical team. The remaining three patients (two with TLE and one with FLE) were evaluated and are currently awaiting surgical intervention.

Additionally, a control group of 19 subjects (14 females and five males; mean age 34.2 years, SD 11.9) with no clinical or imaging evidence of neurological and/or psychiatric disease was also formed.

### Variables

The analyzed variables were sex, age, epilepsy time course, epilepsy type, ictal onset zone laterality, ictal onset zone location, presence of lesions in presurgical brain MRI, presurgical AFQ, surgical technique performed, presence of lesion in postsurgical MRI, postsurgical AFQ, biopsy finding, and postsurgical clinical outcome.

### Measurements of the main results

Patients were evaluated in accordance with the presurgical multimodal evaluation protocol approved by CIREN. It encompasses studies such as prolonged video-electroencephalography monitoring and brain MRI with anatomical and volumetric assessment, among others.

Following surgical intervention and subsequent discharge from the hospital, patients were scheduled for follow-up consultations with a multidisciplinary team to assess postsurgical outcomes and treatment effectiveness. This evaluation entailed a comprehensive physical and neurological examination, brain MRI with anatomical and volumetric studies, electroencephalography (EEG), and other studies.

Postsurgical clinical outcome was determined 1 year after surgery using the modified Engel epilepsy surgery outcome scale (Engel *et al*., 1993). Patients included in Class I of the Engel scale were considered “seizure freedom,” while those included in Classes II–IV of the same scale were considered “seizure recurrence.”

The primary source of clinical data was the medical record of each patient.

### MRI acquisition

All patients underwent brain MRI scans, before and 6 months after surgery, using a 1.5T scanner (Magnetom Aera, Siemens) available at CIREN in Havana. The recommendations for the use of structural MRI in the care of patients with epilepsy were followed (Bernasconi *et al*., 2019).

The parameters used were as follows: T1-MPRAGE 3D sagittal image acquisition, repetition time (RT) = 2200 ms, echo time (ET) = 2.67 ms, inversion time (IT) = 900 ms, 1 mm slice thickness, T2-SPACE FLAIR 3D (RT = 5000 ms, ET = 335 ms, IT = 1800 ms and 1 mm slice thickness), and T2-SPACE (RT = 3200 ms, ET = 381 ms, flip angle = 120 and 1 mm slice thickness). Also, axial isovolumetric SWI sequence and diffusion imaging were included (RT = 9500 ms, ET = 98 ms, matrix size: 128 × 128, 2 mm slice thickness).

The same parameters were used for the MRI study in each individual in the control group.

### MRI analysis

The diffusion images were pre-processed with FSL software (FMRIB Software Library v5.0; Jenkinson *et al*., 2012) The following steps were undertaken: the T1 and DWI sequences were converted from DICOM to NIFTI format. The neck portion of the T1 sequences was removed, and the images were aligned to the anterior commissure–posterior commissure plane, with the anterior commissure serving as the reference point. Brain extraction was performed on the T1 sequence images. The DWI sequences underwent corrections for movement and Eddy currents. Subsequently, the diffusion tensors were corrected, and a DT6.mat file was generated for processing by AFQ.

### Methodology for automated fiber quantification

Once the FSL pre-processing was complete, the tracts were quantified automatically using AFQ software (Yeatman *et al*., 2012).

The AFQ program is a primarily three-step procedure for identifying cerebral fiber tracts: (i) fiber tractography, (ii) waypoint region of interest-based fiber tract segmentation, and (iii) fiber tract refinement based on a probabilistic fiber tract atlas. Further details on the AFQ processing procedure can be found in Yeatman *et al*. (2012).

Of the total of 11 tracts identified by the program, the following bundles were selected based on their role in FLE and TLE seizure propagation: IFOF, TR, CFMe, CH, ILF, and Un (Campos *et al*., 2015; Keller *et al*., 2017; Li *et al*., 2018; da Silva *et al*., 2020; Lin *et al*., 2020).

Subsequently, the characteristics of the white matter tracts of the patients were classified as either “ipsilateral” or “contralateral” to the ictal onset zone (except CFMe). The right and left side characteristics were combined for the controls (Supplementary Fig. 1).

Additionally, the selected tracts were classified into associative (IFOF, CH, ILF, Un), commissural (CFMe), and projection (TR) tracts, according to the anatomical classification system based on their connectivity and trajectory (Carpenter, 1994).

Using AFQ, each tract was divided into 100 equal parts (nodes), in which the following quantitative variables were determined: number of fibers (Fib), volume (Vol), fractional anisotropy (FA), and mean diffusivity (MD).

In this study, five groups (segments) were selected in each tract, each consisting of 20 consecutive nodes. The value of the AFQ variables was averaged in each segment for subsequent statistical processing. Fib was determined globally in each tract, while the remaining AFQ variables were determined globally and segmentally.

Tracts in which AFQ variables could not be measured were discarded.

### Statistical processing

The data were collected and processed using the STATISTICA version 7 program. The following indicators were used at the descriptive statistical level: absolute frequency, mean, and standard deviation. The results of the variables are presented in tabular and graphical formats.

Due to the limited size of the two groups of patients evaluated and the variability in age at the time of evaluation and epilepsy evolution time, it was decided to perform a Kruskal–Wallis test to detect statistically significant differences (*P* < 0.05) in these variables between the two groups.

In contrast, the differences between AFQ-determined variables in the white matter tracts of TLE and FLE patients and controls were assessed using a default ANOVA/ANCOVA general linear model (MANOVA/MANCOVA) and Fisher's least significant difference (LSD) post-hoc test for honest differences.

In addition, a similar analysis was applied to evaluate the differences between the AFQ variables in the white matter tracts of patients with seizure freedom and patients with seizure recurrence 1 year after epilepsy surgery. In all cases, *P* < 0.05 was used as a significant value, with a 95% confidence interval. The results of the analysis are presented in tabular and graphical formats.

In this study, those tracts with significant differences on three or four AFQ variables across their entire length relative to controls were arbitrarily defined as tracts with “consistent global abnormalities.” In addition, tracts with “consistent segmental abnormalities” were those with significant differences in two or three AFQ variables across up to four out of a total of five segments when compared to the control group.

Similarly, it was also arbitrarily determined that in the post-surgical period, tracts with “consistent *de novo* global abnormalities” were those in which significant differences were found in three or four AFQ variables along their entire length. These differences were not evident before surgery compared to the control group.

Moreover, tracts with “consistent *de novo* segmental abnormalities” were identified as those with significant differences in two or three AFQ variables across up to four out of a total of five segments, not evident before surgery compared to controls.

On the other hand, it was decided that “tracts with consistent reinforcement of presurgical global abnormalities” were those in which significant presurgical global differences in three or four AFQ variables persisted to their full extent in the postoperative period compared to the control group.

Furthermore, the “tracts with consistent reinforcement of presurgical segmental abnormalities” were those which demonstrated postsurgical persistence of significant differences in two or three AFQ variables in up to four of five segments, when compared to the control group.

### Ethics

The CIREN ethical committee approved this research. Written informed consent was established and signed by each patient, the legal guardians of the adolescents, and the controls. The single medical team responsible for this study collected information from clinical histories and complementary studies. Furthermore, a unique number was assigned to each patient and control for MRI studies and database identification.

Therefore, no data relating to patient or control identities were disclosed during processing of the information and article writing. Consequently, the ethical parameters defined by the *Declaration of Helsinki* and the national regulations established in the Republic of Cuba were complied with.

## Results

### Characteristics of patients with drug-resistant focal epilepsies

Of the 20 patients studied, eight (40%) had FLE and 12 (60%) had TLE. The mean age in the FLE group was 22 ± 5 years, and in the TLE group it was 33.6 ± 13.8 years. A significant statistical difference was observed between the two groups [Kruskal–Wallis H (1, *n* = 20) = 4.202, *P* = 0.040].

A long epilepsy time course was notable, at an average of 13.4 ± 8.3 years in FLE cases and 17.6 ± 9.7 years in TLE patients. However, no statistically significant difference was identified between the two groups [Kruskal–Wallis H (1, *n* = 20) =0.725, *P* = 0.395].

Of the 17 patients undergoing surgery, complete resection of the affected area was confirmed on postsurgical brain MRI in six patients with FLE (35.7%) and seven with TLE (70%).

Furthermore, on the postoperative clinical evaluation performed 1 year after epilepsy surgery, seizure freedom was observed in six patients with TLE (60%); by contrast, seizure recurrence was detected in six patients with FLE (85.7%).

An additional breakdown of the information on clinical–demographic, electrophysiology, imaging, surgery, biopsy findings, and postoperative outcome characterization of the studied patients is summarized in Supplementary Tables 1 and 2.

### Presurgical white matter anomalies in patients with drug-resistant focal epilepsies

Presurgical white matter tract global and segmental quantitative significant differences between patients and controls are illustrated in Supplementary Tables 3 and 4 respectively. A total of 34 global and 25 segmental anomalies were observed in both ipsilateral and contralateral areas to the ictal onset zone.

**Figure 1:**
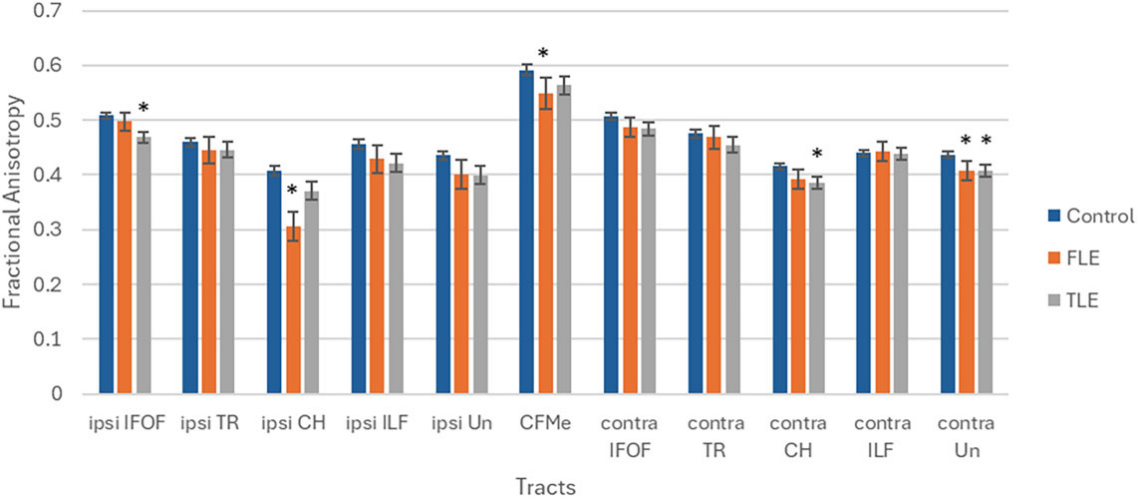
Global fractional anisotropy of white matter tracts in patients who were candidates for surgery, relative to controls.

*Source*: AFQ-MATLAB. Tracts with significant differences showed a decreased global fractional anisotropy in the patient cohort compared to controls. Asterisks (*) represent significant *P* values (<0.05) obtained by general linear model statistical processing. Abbreviations: FLE, frontal lobe epilepsy; TLE, temporal lobe epilepsy. IFOF, inferior fronto-occipital fasciculus; TR, thalamic radiation; CFMe, forceps minor of corpus callosum; CH, cingulum hippocampus; ILF, inferior longitudinal fasciculus; Un, uncinate fasciculus. Ipsi, ipsilateral to ictal onset zone; contra, contralateral to ictal onset zone.

Significant global abnormalities comprised increased MD and reduced FA, Vol, and Fib (Fig. 1 and Supplementary Graphics 1–3). In addition, significant segmental anomalies also consisted of increased MD and reduced FA and Vol.

Significant global differences were identified predominantly in the FA on 13 occasions (11 associative and two commissural tracts). In contrast, significant segmental differences were primarily identified in the MD on 11 occasions (nine associative and two projection tracts).

In addition, global abnormalities exclusive to FLE patients were observed in the MD of the ipsilateral CH and Vol of the ILF. Moreover, the segmental MD of the contralateral ILF was also only affected in patients with FLE.

On the other hand, anomalies in the global MD of the ipsilateral IFOF, Vol of the contralateral IFOF, and segmental MD of the contralateral Un were only detected in patients with TLE.

**Figure 2:**
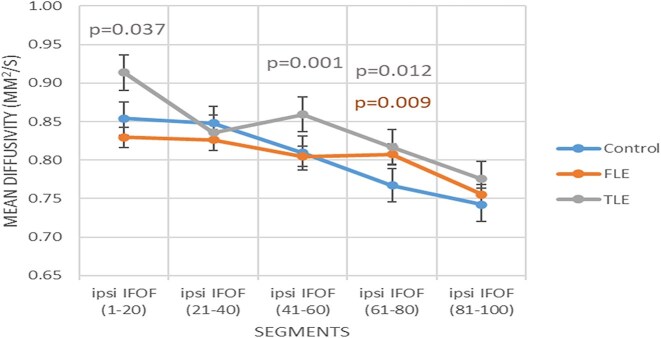
Segmental mean diffusivity of the presurgical ipsilateral inferior fronto-occipital fasciculus in both patient groups compared to controls.

*Source*: AFQ-MATLAB. TLE patients presented a notable elevation (*P* values in grey) in mean diffusivity in segment 1 (nodes 1–20), 3 (nodes 41–60), and 4 (nodes 61–80) when compared to the control group. Furthermore, FLE revealed a significant elevation (*P* values in brown) of the MD in segment 4 (nodes 61–80) when compared with the control group. Abbreviations: IFOF, inferior fronto-occipital fasciculus; Ipsi, ipsilateral to ictal onset zone; TLE, temporal lobe epilepsy.

Both groups of patients exhibited common global abnormalities in FA of the contralateral Un. Furthermore, segmental anomalies in FA of the contralateral ILF, MD of the ipsilateral IFOF (Fig. 2), and the CH were also commonly observed in patients with FLE and TLE.

Moreover, both patient groups revealed consistent global abnormalities in the CFMe and the ipsilateral CH, with FA, Vol, and Fib as the most affected variables. In addition, consistent segmental abnormalities were found in the ipsilateral TR, CH, and ILF and the contralateral TR, with FA and Vol being the most affected variables.

In contrast, no consistent global abnormalities were identified when the FLE and TLE patient groups were evaluated independently. However, FLE patients revealed consistent segmental abnormalities in the ipsilateral CH, contralateral TR and ILF, with MD identified as the most affected variable. Furthermore, the ipsilateral ILF exhibited consistent segmental anomalies in MD and Vol in patients with TLE.

### Postsurgical white matter anomalies in patients with drug-resistant focal epilepsies

Supplementary Tables 5 and 6 show the white matter tracts with significant postsurgical global and segmental quantitative differences between patients and controls respectively. A total of 61 global and 34 segmental abnormalities were identified, located both ipsilateral and contralateral to the ictal onset zone.

**Figure 3:**
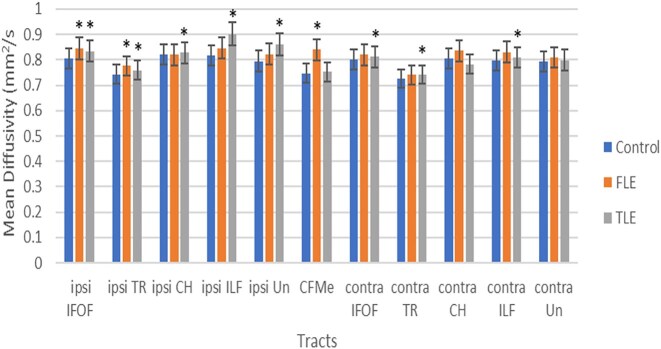
Global mean diffusivity of white matter tracts in patients undergoing surgery, relative to controls.

*Source*: AFQ-MATLAB. Tracts with significant differences showed an increased mean diffusivity in the patient cohort compared to controls. Asterisks (*) represent significant *P* values (<0.05) obtained by general linear model statistical processing. Abbreviations: FLE, frontal lobe epilepsy; TLE, temporal lobe epilepsy. IFOF, inferior fronto-occipital fasciculus; TR, thalamic radiation; CFMe, forceps minor of corpus callosum; CH, cingulum hippocampus; ILF, inferior longitudinal fasciculus; Un, uncinate fasciculus. Ipsi, ipsilateral to ictal onset zone; contra, contralateral to ictal onset zone.

Significant postsurgical global abnormalities included increased MD and reduced FA, Vol, and Fib (Fig. 3 and Supplementary Graphics 4–6). Furthermore, significant postsurgical segmental anomalies also encompassed an increased MD and reduced AF and Vol.

Global MD was the most affected, with anomalies identified on 19 occasions (13 associative, two commissural and four projection tracts). Segmental MD was also the most affected, with 15 abnormalities detected (eight associative and seven projection tracts).

**Figure 4:**
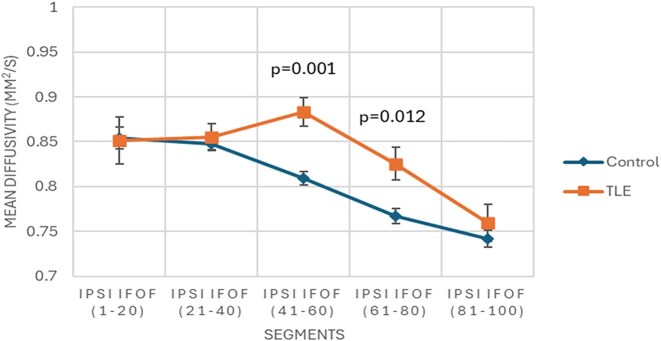
Segmental mean diffusivity of postsurgical ipsilateral inferior fronto-occipital fasciculus in temporal lobe epilepsy patients relative to controls.

*Source*: AFQ-MATLAB. A significant elevation in mean diffusivity is evident in segment 3 (nodes 41–60) and 4 (nodes 61–80) of the tract in the patient cohort when compared to the control group. Abbreviations: IFOF, inferior fronto-occipital fasciculus; Ipsi, ipsilateral to ictal onset zone; TLE, temporal lobe epilepsy.

Furthermore, global anomalies exclusive of TLE patients were observed in the FA of the ipsilateral TR and CH, MD of the ipsilateral CH, and Vol of the ipsilateral Un. Likewise, segmental abnormalities exclusive of TLE patients were observed in the FA of the ipsilateral IFOF, TR, and Un, in addition to the contralateral ILF and Un; MD of the ipsilateral IFOF (Fig. 4) and contralateral Un; and Vol of the ipsilateral IFOF, TR, and Un.

Conversely, no tracts exhibited global or segmental abnormalities exclusively present in FLE patients.

In both patient groups, abnormalities were detected in the FA of the CFMe, the ipsilateral IFOF, and Un; MD of the ipsilateral IFOF and TR; Vol of the CFMe and contralateral CH; and Fib of the contralateral CH. Furthermore, the segmental MD of the ipsilateral TR (Supplementary Graphic 7), Un, and contralateral TR was commonly affected in all patients.

In addition, the ipsilateral TR, ILF and Un, the contralateral CH, and the CFMe showed consistent global abnormalities in both groups of patients. In all cases, Fib was the most affected variable. The ipsilateral CH and ILF, as well as the contralateral TR and CH, revealed consistent segmental abnormalities. In these tracts, MD and Vol were identified as the most affected variables.

In TLE patients, consistent global abnormalities were revealed in the ipsilateral TR, ILF, and Un, in addition to the contralateral CH. In these tracts, Vol and Fib were the most affected variables. Furthermore, consistent segmental abnormalities were observed in the ipsilateral IFOF, TR, ILF, and Un, as well as the contralateral TR, CH, and Un. In these tracts MD was the most affected variable.

In contrast, patients with FLE had consistent global abnormalities in all the variables of the CFMe. However, no tract with consistent segmental abnormalities was observed in this group.

Additionally, a total of 39 *de novo* global anomalies were detected, with MD as the most affected variable on 16 occasions (10 associative, two commissural, and four projection tracts). Moreover, *de novo* segmental alterations were observed on 21 occasions, with MD as the most affected variable on nine occasions (five associative and five projection tracts).

Furthermore, the ipsilateral ILF was the tract with *de novo* global anomalies irrespective of the epilepsy type, with involvement of MD, Vol, and Fib. Similarly, both patient groups showed consistent *de novo* segmental abnormalities in MD and Vol of the contralateral CH.

Additionally, FLE patients demonstrated consistent *de novo* global abnormalities in the CFMe, with MD, Vol and Fib as the affected variables. However, no tracts exhibited consistent *de novo* segmental abnormalities in this group.

TLE patients revealed consistent *de novo* global anomalies in Vol and Fib of the ipsilateral ILF and Un. In addition, consistent *de novo* segmental anomalies were observed in FA of the ipsilateral IFOF and the contralateral CH, as well as the ipsilateral and contralateral TR and Un.

Conversely, the presence of tracts exhibiting a reinforcement of their global presurgical abnormalities in the postsurgical period was documented on 21 occasions. This phenomenon was predominantly identified in FA on nine occasions (seven associative and two commissural tracts).

Furthermore, the presence of tracts whose presurgical segmental abnormalities were reinforced in the postsurgical period was observed on 11 occasions, with MD as the variable with the highest number of anomalies (three associative and two projection tracts).

In both patient groups, postsurgical reinforcement of the presurgical global abnormalities was consistently observed in FA, Vol, and Fib of the CFMe. Moreover, consistent postsurgical reinforcement of presurgical segmental abnormalities was evidenced in FA, MD, and Vol of the ipsilateral CH and ILF, as well as the contralateral TR.

In contrast, no consistent postsurgical reinforcement of presurgical global abnormalities dependent on the type of epilepsy was observed.

Nevertheless, patients with TLE showed consistent postsurgical reinforcement of the presurgical segmental abnormalities in MD and Vol of the ipsilateral ILF. However, in FLE patients, no consistent postsurgical reinforcement of presurgical segmental anomalies was evident.

### Presurgical white matter anomalies and clinical outcome

Supplementary Table 7 shows the cerebral white matter tracts with significant presurgical global and segmental quantitative differences between FLE and TLE patients concerning their clinical outcome 1 year after surgery.

The global MD of the contralateral ILF and Un revealed significant differences in all patients. Similarly, TLE patients presented an increased MD in both tracts in patients with seizure freedom compared to those with seizure recurrence (Supplementary Graphic 8).

In contrast, no preoperative global abnormalities were found between FLE patients with seizure freedom and seizure recurrence 1 year after surgery.

**Figure 5:**
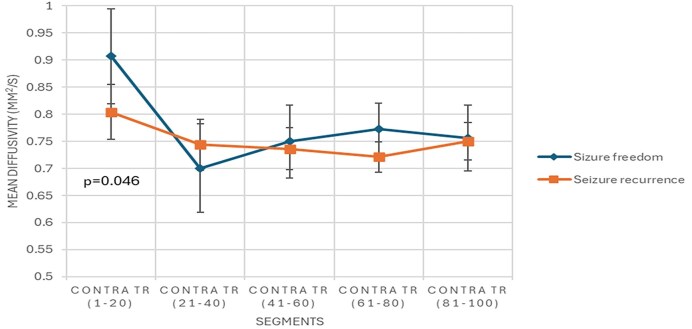
Presurgical segmental mean diffusivity of the contralateral thalamic radiation in patients with drug-resistant focal epilepsy and their postsurgical clinical outcome.

*Source*: AFQ-MATLAB. Patients with postsurgical seizure freedom had a significant increase in presurgical mean diffusivity of segment 1 (nodes 1–20) compared to patients with seizure recurrence. Abbreviations: TR, thalamic radiation; Contra, contralateral to epileptogenic zone.

Both groups of patients showed a significant increase in MD of segment 1 (nodes 1–20) of the contralateral TR in those with seizure freedom compared to those with seizure recurrence (Fig. 5).

However, in independent analysis of the FLE and TLE patient groups, no presurgical segmental abnormalities were observed related to postoperative clinical outcome.

### Postsurgical cerebral white matter anomalies and clinical outcome

**Figure 6:**
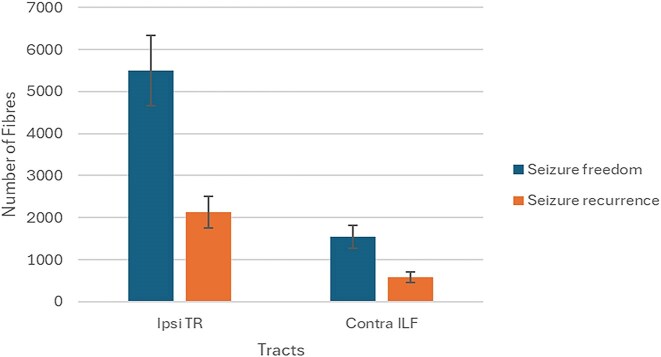
Postsurgical number of fibers of the ipsilateral thalamic radiation and contralateral inferior longitudinal fasciculus in patients with frontal lobe epilepsy and their postsurgical clinical outcome.

*Source*: AFQ-MATLAB. Patients with postsurgical seizure freedom had a significant increase in the postsurgical number of fibers of the ipsilateral thalamic radiation and contralateral inferior longitudinal fasciculus compared to patients with seizure recurrence. Abbreviations: TR, thalamic radiation; ILF, inferior longitudinal fasciculus; Ipsi, ipsilateral to epileptic zone; contra, contralateral epileptic zone.

Supplementary Table 8 illustrates the cerebral white matter tracts with significant postsurgical global quantitative differences between FLE and TLE patients concerning their clinical outcome 1 year after surgery. Fib of the ipsilateral TR and the contralateral ILF differed significantly in the FLE group. Patients with seizure freedom had the highest values of this variable in both tracts (Fig. 6).

Furthermore, the TLE group exhibited significant global quantitative differences in MD of the ipsilateral IFOF, with lower values observed in patients exhibiting seizure freedom compared to those with seizure recurrence (Supplementary Graphic 9).

In addition, Supplementary Table 9 shows the cerebral white matter tracts with significant postsurgical segmental quantitative differences between FLE and TLE patients concerning their clinical outcome 1 year after surgery.

Patients with FLE had significant differences in the segmental MD of the CFMe (Supplementary Graphic 10) and contralateral CH. Additionally, the segmental Vol of the ipsilateral ILF and the contralateral IFOF and Un (Supplementary Graphic 11) showed significant discrepancies in this group of patients.

Moreover, patients with TLE showed significant differences in the segmental FA of contralateral TR, segmental MD of the ipsilateral Un, and segmental Vol of the ipsilateral IFOF and TR.

All patients had significant segmental differences in FA of the contralateral TR.

## Discussion

The application of AFQ enabled us to identify preoperative and postoperative anomalies situated along or within particular segments of the commissural, associative, or projection white matter tracts, located both ipsilateral and contralateral to the epileptogenic zone in patients presenting with focal DRE. These abnormalities may be specific to patients with FLE or TLE, or they may be common to both types of epilepsy. Furthermore, these abnormalities may also vary regarding postoperative clinical outcome.

### Presurgical white matter abnormalities in patients with drug-resistant focal epilepsies

We observed consistent global abnormalities in the CFMe and ipsilateral CH in both patient groups. Furthermore, we also found consistent segmental anomalies in the ipsilateral TR, CH, and ILF, as well as in the contralateral TR.

In patients with FLE, consistent segmental anomalies were observed in the ipsilateral CH, contralateral TR, and ILF. In addition, there was evidence of consistent segmental abnormalities in the ipsilateral ILF in patients with TLE.

Our results add more evidence to the already known neural network hypothesis as one of the underlying mechanisms of DRE. This hypothesis postulates that seizure-induced neuronal loss and axonal damage may lead to reorganization of the neuronal network, which can result in a high tendency for clustering of connections between adjacent structures. This, in turn, increases excitability, epileptogenicity, and seizure recurrence (Fang *et al*., 2011; Kreilkamp *et al*., 2021).

As observed, epilepsy is a network disorder, encompassing structural alterations that extend beyond the epileptogenic zone (EZ) (Whelan *et al*., 2018; Hall *et al*., 2023). In the case of TLE and FLE, several white matter tracts have been described as having an important role in the dissemination of epileptic activity. This is the case of the Un, IFOF, ILF, TR, CFMe, and CH (Campos *et al*., 2015; Keller *et al*., 2017).

Our data coincide with a previous AFQ study (Keller *et al*., 2017) which evidenced anomalies confined to specific regions or spanning the entire length of white matter tracts in both hemispheres, such as the Un.

For instance, bilateral white matter anomalies have also been observed in patients with drug-resistant TLE and FLE using diverse imaging processing methodologies (Campos *et al*., 2015; Glenn *et al*., 2016; Lin *et al*., 2020; Urquia-Osorio *et al*., 2022). This type of abnormality has been detected in tracts such as the TR and ILF in FLE patients and the ILF in TLE (Lin *et al*., 2020). This information is consistent with the segmental abnormalities identified in FLE and TLE patients in our investigation.

Furthermore, there is evidence of diffusional abnormalities in tracts such as the ipsilateral TR, CH, and ILF, as well as the contralateral TR and CFMe, in patients with TLE and FLE (Lin *et al*., 2020). These data are also in agreement with the consistent global and segmental abnormalities observed in the combined analysis of patients with FLE and TLE in our investigation.

Furthermore, previous research has indicated that the brain damage experienced by TLE patients is more likely to involve structures within the limbic system, particularly regions that are anatomically and functionally related to the hippocampus. These regions include the entorhinal and perirhinal cortex, the thalamus, the anterior cingulate, and the cortical association areas (Bonilha *et al*., 2012).

In contrast, bilateral diffusion anomalies of the IFOF, Un and fornix have been detected in TLE patients. Additionally, bilateral abnormalities in the cingulum have also been observed in FLE patients (Campos *et al*., 2015).

This information does not coincide with the tracts showing consistent segmental abnormalities in TLE and FLE patients in our research.

It is important to note that the aforementioned studies employed disparate image processing techniques, which did not encompass all of the tracts investigated in our research. Furthermore, the studies included patients with specific etiologies of the DRE, whereas our research encompassed a wider spectrum of causes of epilepsy.

However, the preceding data, whether discrepant or coincidental, collectively support the assertion that the location of the epileptogenic network and the underlying cause of epilepsy may be associated with the extent of white matter involvement in the patients (Campos *et al*., 2015).

Additionally, our research indicated that the quantitative white matter modifications predominantly comprised a reduction in FA, Vol, and Fib, accompanied by an increase in MD in the tracts of patients with DRE in comparison to controls.

We observed that in patients with FLE and TLE, the most affected variables were FA, Vol, and Fib in tracts with consistent global abnormalities, and FA and Vol in tracts with consistent segmental abnormalities. Furthermore, the most affected variable in tracts with consistent segmental abnormalities was MD in patients with FLE, and Vol and MD in patients with TLE.

In line with the consistent segmental abnormalities identified in TLE patients in our study, previous research has indicated that pathological variations in MD are more closely associated with potential epileptogenic tissue (Keller *et al*., 2017).

In addition, the findings of another study indicated that FA was the most affected variable in patients with FLE and TLE (Lin *et al*., 2020), which aligns with the observations made in our research.

### Postsurgical white matter abnormalities in patients with drug-resistant focal epilepsies

In the combined evaluation of patients with FLE and TLE, we observed tracts with consistent postsurgical global abnormalities, including the CFMe, the ipsilateral TR, ILF and Un, in addition to the contralateral CH. Meanwhile, the tracts with consistent segmental abnormalities were the ipsilateral CH and ILF, as well as the contralateral TR and CH.

The CFMe exhibited consistent global abnormalities in patients with FLE. Conversely, consistent segmental anomalies were not observed in this group. In addition, TLE patients revealed consistent postsurgical global abnormalities in the ipsilateral TR, ILF, and Un, in addition to the contralateral CH. Moreover, the tracts with consistent segmental abnormalities were the ipsilateral IFOF, TR, ILF, and Un, as well as the contralateral TR, CH ,and Un.

One of the goals of epilepsy surgery is control of the disease. To achieve this, several techniques are used to resect, disconnect, or modulate the EZ (Hodelín Mainard *et al*., 2021). As a consequence, it is obvious to expect an anatomical and functional modification of the remaining tissue.

Previous scientific evidence has confirmed the existence of Wallerian degeneration after temporal lobectomy in brain regions connected to a greater or lesser extent to the temporal lobe (Yogarajah *et al*., 2010). These findings are in line with the bilateral white matter abnormalities that have been identified in TLE patients in our research.

Furthermore, research has confirmed the presence of postsurgical connectivity abnormalities in areas in close relation to the resected tissue in TLE patients, including the amygdala, hippocampus, parahippocampal gyrus, and temporal pole ipsilateral to the EZ (Taylor *et al*., 2018). However, in contrast to the findings of our previous research, no contralateral abnormalities were observed in this study.

Nevertheless, in accordance with the bilateral segmental findings detected in our research, previous evidence of bilateral anomalies has also been detected in areas such as the superior part of the TR (Bartoňová *et al*., 2023).

Furthermore, our investigation revealed that postsurgical quantitative abnormalities were characterized by a reduction in FA, Vol, and Fib, in addition to an increase in MD.

In the combined evaluation of patients with FLE and TLE, we observed that Fib was the most affected variable in tracts with consistent global abnormalities, whereas MD and Vol were most frequently affected in tracts with consistent segmental anomalies. In TLE patients, Vol and Fib were the most affected in tracts with consistent global anomalies, while MD was the most affected variable in tracts with consistent segmental abnormalities. Furthermore, the only tract displaying consistent global anomalies in FLE patients had affected all the quantitative variables.

Previous evidence confirmed that after epilepsy surgery, the short path connections contained in the resected tissue can change their direction, traversing other areas, and increasing or decreasing in number (da Silva *et al*., 2020).

Moreover, it is also known that the white matter transected during surgery can lead to myelin degeneration and axonal bundle reduction, in addition to Wallerian degeneration. These abnormalities are reflected in the AF changes of the tracts in the first month after surgery (da Silva *et al*., 2020), which aligns with the findings of our research.

Other mechanisms may also provide a rationale for the variation of FA in patients following epilepsy surgery. These include increased myelination, axonal diameter, fibre density or changes in the structure of axonal membranes (Moore *et al*., 2017).

Furthermore, similar to the results of our study, previous research also confirmed the reduction of Vol and Fib in areas related to surgical resection in patients with TLE undergoing epilepsy surgery (Taylor *et al*., 2018).

### Presurgical and postsurgical anomalies and clinical outcome in patients with drug-resistant focal epilepsies

In our study, we observed that 60% of TLE patients and 14.3% of the FLE patients were seizure-free 1 year after surgery.

Moreover, the pooled analysis revealed that seizure-free patients exhibited an increased preoperative global MD of the contralateral ILF and Un, and an increased preoperative segmental MD of the contralateral TR compared to patients who experienced seizure recurrence. Similarly, an increased preoperative global MD in the contralateral ILF and Un was observed in TLE seizure-free patients compared to those with seizure recurrence.

In agreement with our findings, previous research revealed presurgical MD abnormalities related to the postsurgical outcome in circumscribed regions of the parahippocampal white matter contralateral to the EZ in TLE patients (Keller *et al*., 2017). Contrasting with our findings, this study also evidenced segmental abnormalities in the FA of the Un ipsilateral to the EZ in TLE patients with postsurgical seizure recurrence (Keller *et al*., 2017).

Furthermore, our findings align with the observation of an increase in MD in TLE patients who have achieved postsurgical seizure freedom. This increase has been noted in tracts such as the fornix, cingulum, external capsule from both hemispheres and the genu of the corpus callosum (Concha *et al*., 2007). In contrast, a presurgical reduced FA has also been observed in TLE postsurgical seizure-free patients in the ipsilateral posterior arm of the internal capsule, anterior corona radiata, as well as contralateral optic radiation (Li *et al*., 2018).

In the cohort of patients with FLE included in our study, no statistically significant presurgical alterations were observed concerning postsurgical clinical outcome. This constitutes a novel contribution to the field as no similar studies were identified in the existing literature.

Furthermore, we detected a postsurgical significant global reduction of MD in the IFOF ipsilateral to EZ in TLE patients with seizure freedom after surgery, compared to those with seizure recurrence. In the group of FLE patients, we detected a postsurgical increase in Fib in the ipsilateral TR and contralateral ILF in patients with seizure freedom relative to those with seizure recurrence.

We also observed postsurgical segmental anomalies in MD of the CFMe and contralateral CH, in addition to Vol abnormalities in the ipsilateral ILF, and contralateral IFOF and Un in patients with FLE. In patients with TLE, segmental anomalies were found in FA of the contralateral TR, MD of ipsilateral Un, and Vol of ipsilateral IFOF and TR. Also, we found segmental anomalies in FA of the contralateral TR, as a result of the combined evaluation of patients with FLE and TLE.

These findings indicate cerebral white matter reorganization in patients with seizure freedom following surgery. Also, they coincide with previous observations that have shown that just as surgery can stop seizures, seizure-induced white matter damage can be reversible (Yasuda *et al*., 2010).

In contrast to the findings of our investigation, it has been described that seizure-free TLE patients have a postsurgical reduction in the quantitative anisotropy (FA-like measurement) of tracts such as the IFOF and ILF in both cerebral hemispheres and in the contralateral TR (da Silva *et al*., 2020), in addition to increased MD and a decreased FA in tracts such as the bilateral fornix, cingulum, external capsule, and splenium of the corpus callosum (Concha *et al*., 2007).

It is important to note that the discrepancies in the findings of the referenced studies in comparison to our research may be attributed to the use of disparate image-processing methodologies in the analysis of the various cerebral white matter tracts.

During a detailed bibliographic review for the present paper, we could not find any evidence of the use of AFQ methodology in postoperative MRI studies of patients with DRE. Consequently, we believe that our research could be the first to demonstrate white matter anomalies using AFQ in epileptic patients undergoing epilepsy surgery.

The evidence obtained in our research shows how global and restricted white matter tract anomalies can vary concerning postsurgical outcome. AFQ's capacity to identify these anomalies has the potential to positively impact clinical practice by offering doctors the ability to identify patients who may benefit from surgical intervention.

However, a cautious interpretation of our data is required due to the small sample size of patients, the heterogeneity of surgical techniques performed, the time of evolution of the DRE, and the age differences in the cohort of patients. These facts limit the generalization of our results.

To address the limitations of our current research, it would be beneficial to consider larger multicenter studies in the future to validate our findings on a larger scale. The findings of these studies could provide a foundation for expanding the clinical use of AFQ as an auxiliary tool for surgical planning. This would include considering both diffuse and restricted white matter abnormalities as potential surgical targets for epilepsy surgery. Furthermore, AFQ has the potential to serve as a valuable tool in predicting clinical outcomes following epilepsy surgery in routine clinical practice.

Finally, another study design that may be considered is the use of AFQ to explore whether patients without DRE have quantitative abnormalities of the cerebral white matter. Should such abnormalities be evident, it would also be possible to determine whether they have prognostic value for the progression to DRE in these patients.

## Conclusions

Patients with drug-resistant epilepsies have preoperative and postoperative global and segmental quantitative abnormalities, which may also vary concerning postsurgical clinical outcome. The postsurgical white matter changes suggest reorganization of the neural network in this disease.

Preoperative increased global mean diffusivity of the contralateral inferior longitudinal fasciculus and uncinate fasciculus was observed in seizure-free TLE patients. Furthermore, DRE patients with seizure freedom had a preoperative increased segmental mean diffusivity in the contralateral thalamic radiation.

Additionally, FLE patients with seizure freedom exhibited postsurgical increases in the number of fibers of the ipsilateral thalamic radiation and contralateral inferior longitudinal fasciculus. Furthermore, TLE patients with seizure freedom had a lower postsurgical global mean diffusivity in the ipsilateral inferior fronto-occipital fasciculus.

These findings show the potential of AFQ as a useful methodology for the presurgical and postsurgical evaluation of patients with drug-resistant epilepsies. Considering its future applications in daily clinical practice could improve the management of this sensitive group of patients.

## Supplementary Material

kkaf015_Supplemental_File
