# Supplementary material for: White matter quantitative anomalies and clinical outcome in drug-resistant epilepsies
Source: Psychoradiology. 2025 May 28;5:kkaf015. doi: 10.1093/psyrad/kkaf015 (PMC12164746; doi:10.1093/psyrad/kkaf015)
Supplement: kkaf015_Supplemental_File [file kkaf015_supplemental_file.pdf]

SUPPLEMENTARY MATERIALS

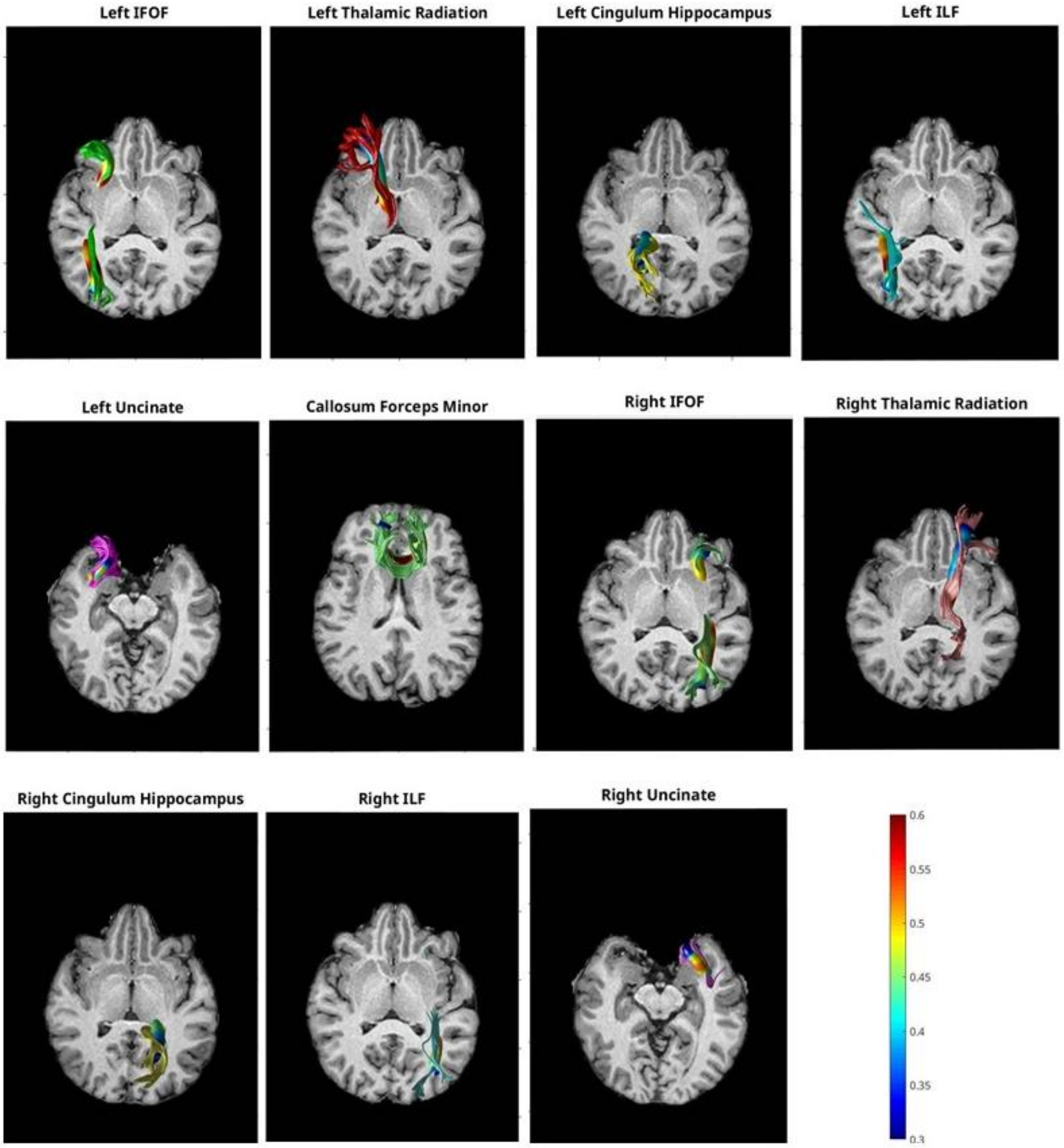

Figure 1. Magnetic resonance T1 axial images obtained in a control, and the example of fractional anisotropy measurement by Automated Fiber Quantification in tracts segments selected for the investigation. Fractional anisotropy values are reflected in the colour scale. ***Abbreviations:*** *IFOF-inferior fronto-occipital fasciculus; ILF-inferior longitudinal fasciculus.*

Graphic 1. Global mean diffusivity of white matter tracts in patients candidates for surgery, relative to controls.

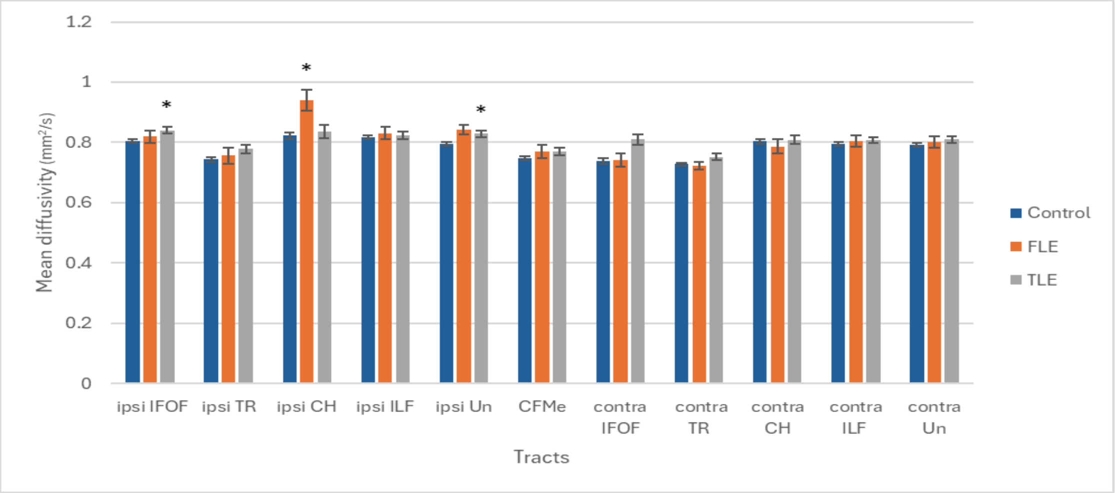

Source: AFQ-MATLAB

Tracts with significant differences showed an increased global mean diffusivity in the patient cohort compared to controls. Asterisks (*) represent significant "p" values (< 0.05) obtained by General Linear Model statistical processing. ***Abbreviations:*** *FLE-frontal lobe epilepsy; TLE- temporal lobe epilepsy. IFOF-Inferior fronto-occipital fasciculus; TR- thalamic radiation; CFMe- forceps minor of corpus callosum; CH- cingulum hippocampus; ILF- inferior longitudinal fasciculus; Un- uncinate fasciculus. Ipsi-ipsilateral to ictal onset zone; contra-contralateral to ictal onset zone.*

Graphic 2. Global volume of white matter tracts in patients candidates for surgery, relative to controls.

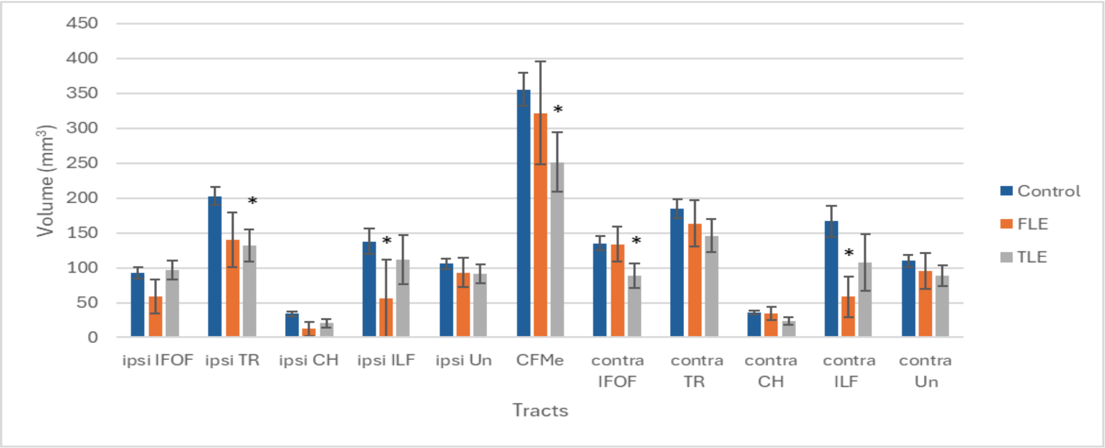

Source: AFQ-MATLAB

Tracts with significant differences showed a decreased global volume in the patient cohort compared to controls. Asterisks (*) represent significant "p" values (< 0.05) obtained by General Linear Model statistical processing. ***Abbreviations:*** *FLE-frontal lobe epilepsy; TLE-temporal lobe epilepsy. IFOF-Inferior fronto-occipital fasciculus; TR- thalamic radiation; CFMe- forceps minor of corpus callosum; CH- cingulum hippocampus; ILF- inferior longitudinal fasciculus; Un- uncinate fasciculus. Ipsi-ipsilateral to ictal onset zone; contra-contralateral to ictal onset zone.*

Graphic 3. Number of fibers of white matter tracts in patients candidates for surgery, relative to controls.

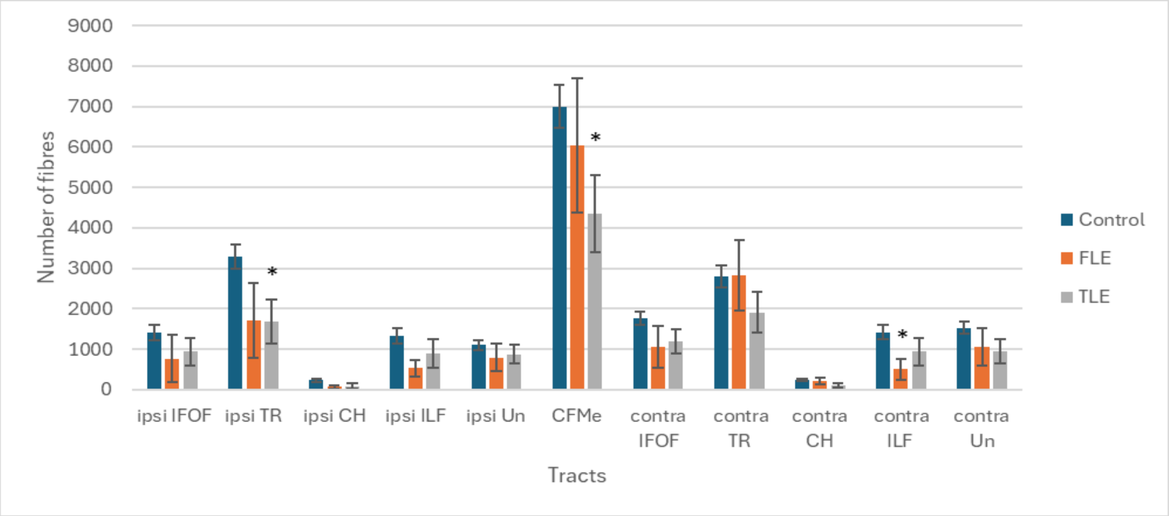

Source: AFQ-MATLAB

Tracts with significant differences showed a decreased number of fibers in the patient cohort compared to controls. Asterisks (*) represent significant "p" values (< 0.05) obtained by General Linear Model statistical processing. ***Abbreviations:*** *FLE-frontal lobe epilepsy; TLE-temporal lobe epilepsy. IFOF-Inferior fronto-occipital fasciculus; TR- thalamic radiation; CFMe- forceps minor of corpus callosum; CH- cingulum hippocampus; ILF- inferior longitudinal fasciculus; Un- uncinate fasciculus. Ipsi-ipsilateral to ictal onset zone; contra-contralateral to ictal onset zone.*

Graphic 4. Global fractional anisotropy of white matter tracts in patients undergoing surgery, relative to controls.

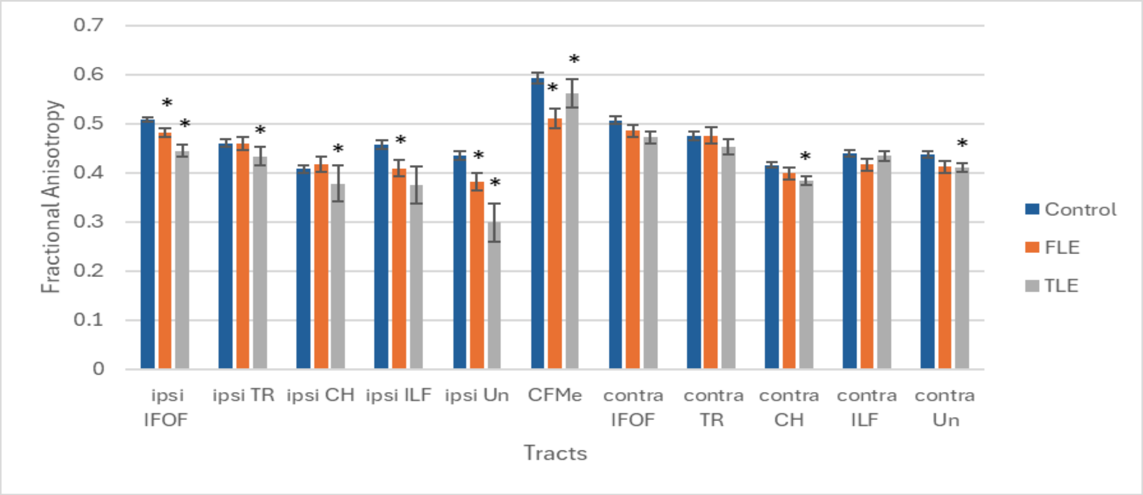

Source: AFQ-MATLAB

Tracts with significant differences showed a decreased global fractional anisotropy in the patient cohort compared to controls. Asterisks (*) represent significant "p" values (< 0.05) obtained by General Linear Model statistical processing. ***Abbreviations:*** *FLE-frontal lobe epilepsy; TLE-temporal lobe epilepsy. IFOF-Inferior fronto-occipital fasciculus; TR-thalamic radiation; CFMe-forceps minor of corpus callosum; CH-cingulum hippocampus; ILF-inferior longitudinal fasciculus; Un-uncinate fasciculus. Ipsi-ipsilateral to ictal onset zone; contra-contralateral to ictal onset zone.*

Graphic 5. Global volume of white matter tracts in patients undergoing surgery, relative to controls.

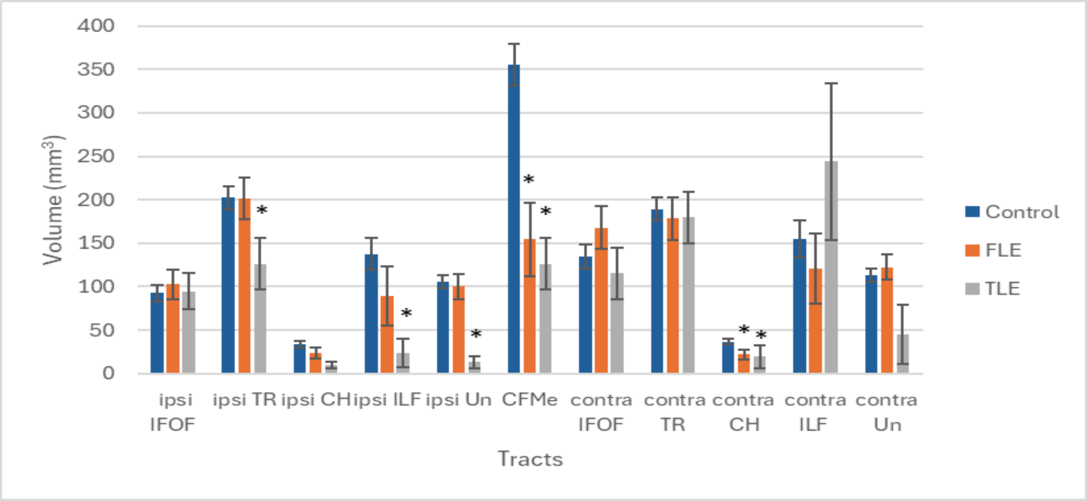

Source: AFQ-MATLAB

Tracts with significant differences showed a decreased global volume in the patient cohort compared to controls. Asterisks (*) represent significant "p" values (< 0.05) obtained by General Linear Model statistical processing. ***Abbreviations:*** *FLE-frontal lobe epilepsy; TLE-temporal lobe epilepsy. IFOF-Inferior fronto-occipital fasciculus; TR- thalamic radiation; CFMe-forceps minor of corpus callosum; CH-cingulum hippocampus; ILF-inferior longitudinal fasciculus; Un-uncinate fasciculus. Ipsi-ipsilateral to ictal onset zone; contra-contralateral to ictal onset zone.*

Graphic 6. Number of fibers of white matter tracts in patients undergoing surgery, relative to controls.

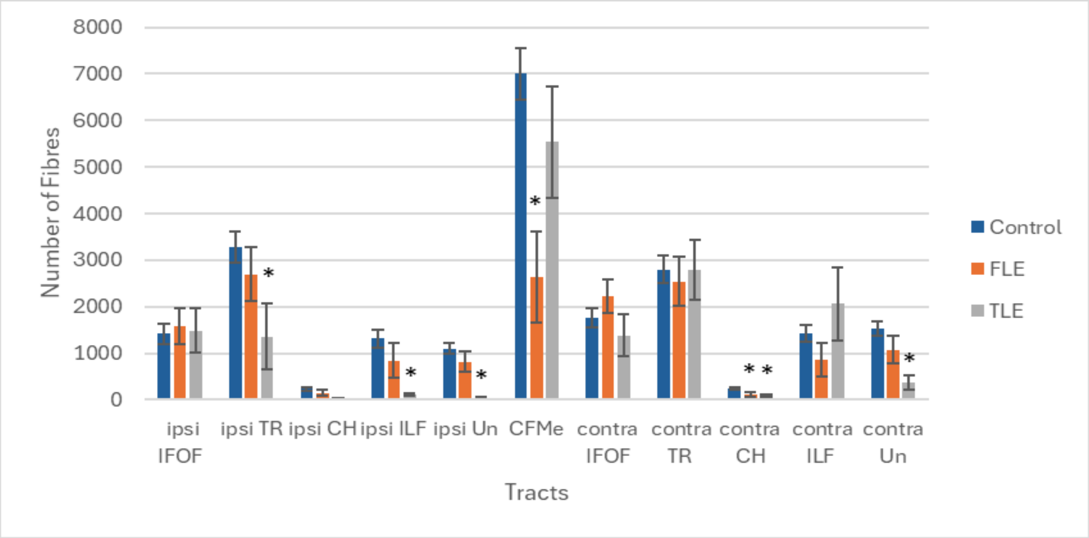

Source: AFQ-MATLAB

Tracts with significant differences showed a decreased number of fibers in the patient cohort compared to controls. Asterisks (*) represent significant "p" values (< 0.05) obtained by General Linear Model statistical processing. ***Abbreviations:*** *FLE-frontal lobe epilepsy; TLE-temporal lobe epilepsy. IFOF-Inferior fronto-occipital fasciculus; TR-thalamic radiation; CFMe-forceps minor of corpus callosum; CH-cingulum hippocampus; ILF-inferior longitudinal fasciculus; Un-uncinate fasciculus. Ipsi-ipsilateral to ictal onset zone; contra-contralateral to ictal onset zone.*

Graphic 7. Segmental mean diffusivity of postsurgical ipsilateral thalamic radiation in both patient groups compared to controls.

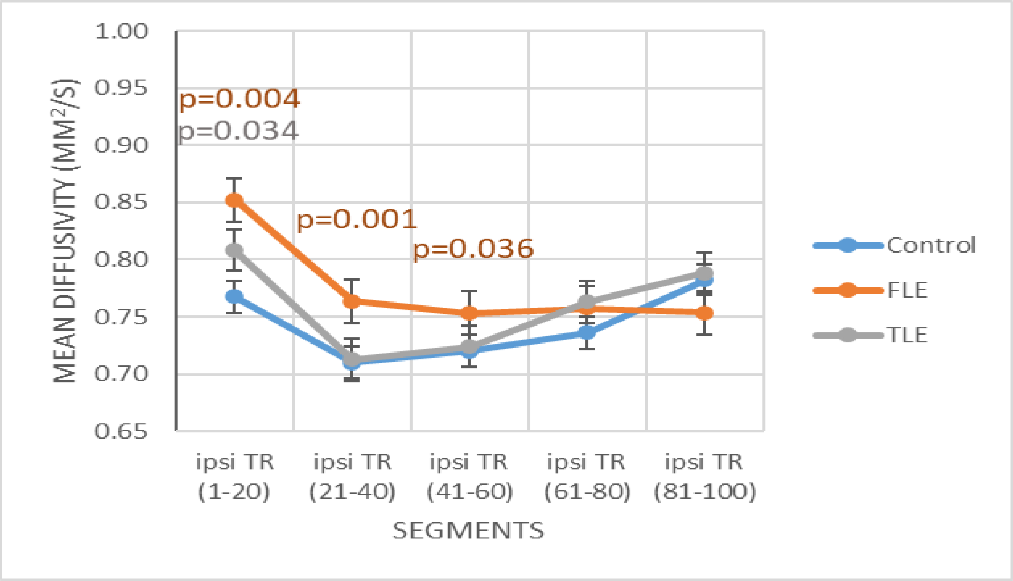

Source: AFQ-MATLAB

TLE patients presented a notable elevation ("p" values in grey colour) in mean diffusivity in segment one (nodes 1-20) when compared to the control group. Furthermore, FLE revealed a significant elevation ("p" values in brown colour) of the MD in segment one (nodes 1-20), two (nodes 21-40) and three (nodes 41-60) when compared with the control group. ***Abbreviation:*** *TR-thalamic radiation; ipsi-ipsilateral to ictal onset zone; FLE-frontal lobe epilepsy.*

Graphic 8. Presurgical global mean diffusivity of the uncinate and inferior longitudinal fasciculus in patients with temporal lobe epilepsy and their postsurgical clinical outcome.

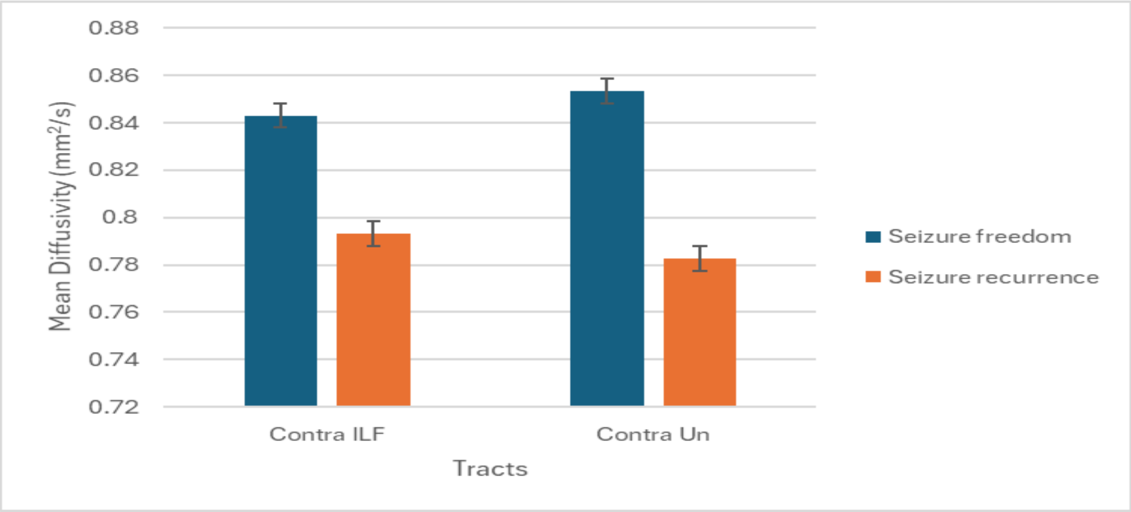

Source: AFQ-MATLAB

Patients with postsurgical seizure freedom had a significant increase in presurgical global mean diffusivity of the contralateral inferior longitudinal fasciculus and uncinate fasciculus compared to patients with seizure recurrence. ***Abbreviations:*** *ILF- inferior longitudinal fasciculus; Un- uncinate fasciculus; Contra- contralateral to ictal onset zone.*

Graphic 9. Postsurgical global mean diffusivity of the ipsilateral inferior fronto-occipital fasciculus in patients with temporal lobe epilepsy and their postsurgical clinical outcome.

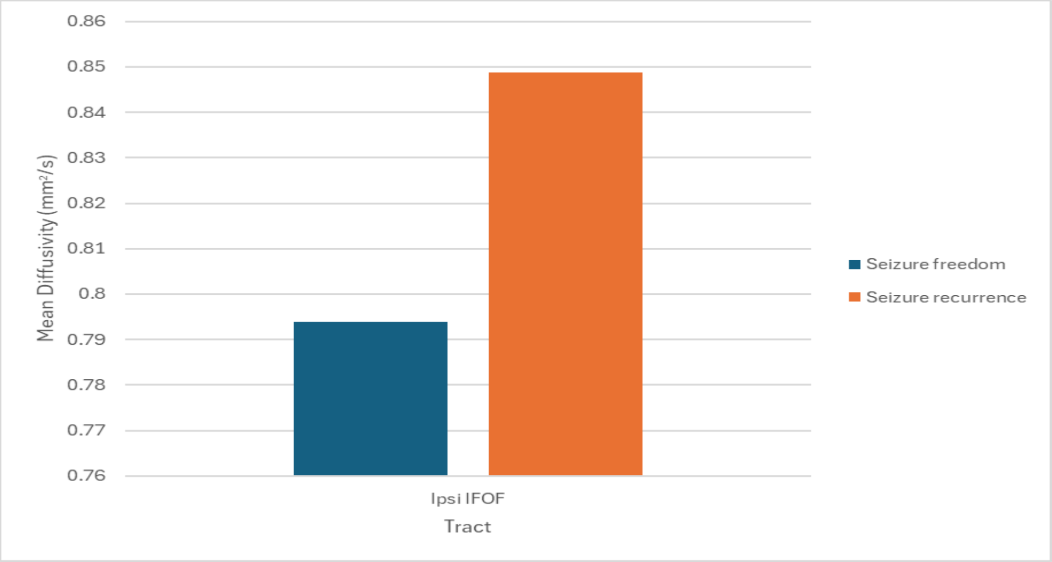

Source: AFQ -MATLAB

Patients with postsurgical seizure freedom had a significant decrease in postsurgical global mean diffusivity of the ipsilateral inferior fronto-occipital fasciculus compared to patients with seizure recurrence. ***Abbreviations:*** *IFOF-inferior fronto-occipital fasciculus. Ipsi-ipsilateral to epileptogenic zone.*

Graphic 10. Segmental mean diffusivity of postsurgical forceps minor of corpus callosum in patients with frontal lobe epilepsy, and their postsurgical clinical outcome.

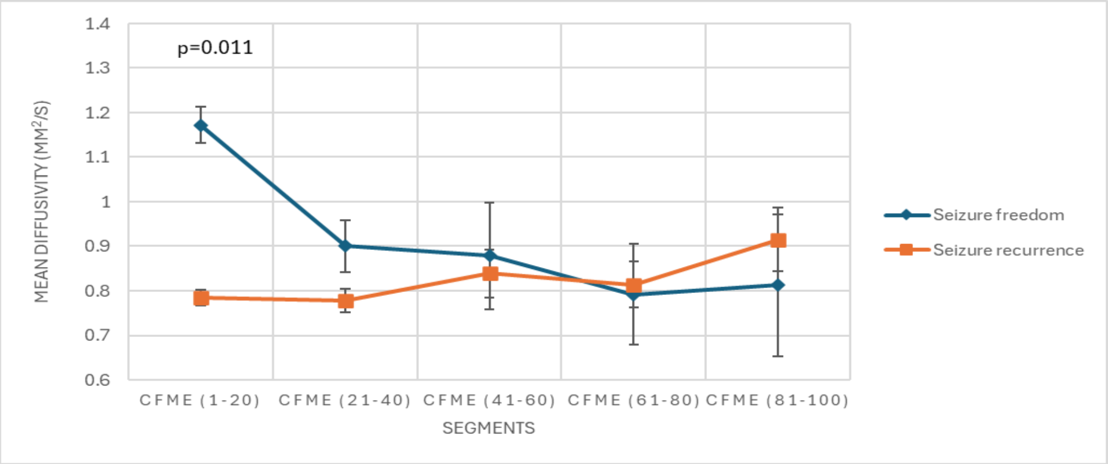

Source: AFQ-MATLAB

Compared to patients with seizure recurrence, patients with postsurgical seizure freedom had a significant increase in postsurgical volume in segment one (nodes 1-20) of the forceps minor of the corpus callosum. ***Abbreviations:*** *CFMe-Forceps minor of corpus callosum.*

Graph 11. Postsurgical segmental volume of the contralateral uncinate fasciculus in patients with frontal lobe epilepsy, and their postsurgical clinical outcome.

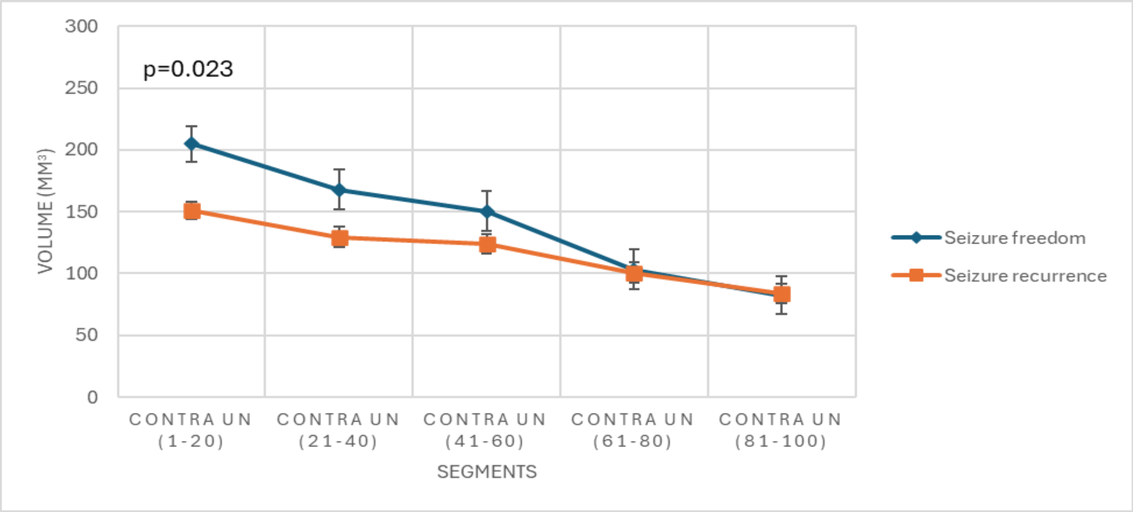

Source: AFQ -MATLAB

Patients with postsurgical seizure freedom had a significant increase in postsurgical volume in segment one (nodes 1-20) of the contralateral uncinate fasciculus compared to patients with seizure recurrence. ***Abbreviations:*** *Un-uncinate fasciculus. Contra-contralateral.*
